# Immunotoxin Against a Donor MHC Class II Molecule Induces Indefinite Survival of Murine Kidney Allografts

**DOI:** 10.1111/ajt.13584

**Published:** 2016-01-22

**Authors:** K. Brown, A. K. Nowocin, L. Meader, L. A. Edwards, R. A. Smith, W. Wong

**Affiliations:** ^1^ Medical Research Council (MRC) Centre for TransplantationSchool of Medicine at Guy's, King's, and St. Thomas' HospitalsKing's College LondonLondonUK

**Keywords:** basic (laboratory) research, science, immunobiology, kidney transplantation, nephrology, animal models, murine, antigen presentation, recognition, dendritic cell, immunosuppressant, fusion proteins and monoclonal antibodies, major histocompatibility complex (MHC)

## Abstract

Rejection of donor organs depends on the trafficking of donor passenger leukocytes to the secondary lymphoid organs of the recipient to elicit an immune response via the direct antigen presentation pathway. Therefore, the depletion of passenger leukocytes may be clinically applicable as a strategy to improve graft survival. Because major histocompatibility complex (MHC) class II^+^ cells are most efficient at inducing immune responses, selective depletion of this population from donor grafts may dampen the alloimmune response and prolong graft survival. In a fully MHC mismatched mouse kidney allograft model, we describe the synthesis of an immunotoxin, consisting of the F(ab′)_2_ fragment of a monoclonal antibody against the donor MHC class II molecule I‐A^k^ conjugated with the plant‐derived ribosomal inactivating protein gelonin. This anti–I‐A^k^ gelonin immunotoxin depletes I‐A^k^ expressing cells specifically *in vitro* and *in vivo*. When given to recipients of kidney allografts, it resulted in indefinite graft survival with normal graft function, presence of Foxp3^+^ cells within donor grafts, diminished donor‐specific antibody formation, and delayed rejection of subsequent donor‐type skin grafts. Strategies aimed at the donor arm of the immune system using agents such as immunotoxins may be a useful adjuvant to existing recipient‐orientated immunosuppression.

## Abstract 2

MHC class II–specific immunotoxin allows long‐term murine kidney allograft survival without apparent protective immune effects.

**Abbreviations**
BUN: blood urea nitrogen
DC: dendritic cell
ELISpot: enzyme‐linked immunospot
EYFP: enhanced yellow fluorescent protein
IFNγ: interferon‐γ
MHC: major histocompatibility complex
TLO: tertiary lymphoid organ

## Introduction

The key initiating event during the rejection of transplanted organs is the interaction between donor passenger leukocytes and recipient T cells. This was first demonstrated by a series of experiments where donor grafts were depleted of passenger leukocytes by being “parked“ in primary recipients under cover of immunosuppression before being retransplanted 1. These grafts demonstrated prolonged survival in the secondary naive recipients, but this beneficial effect was abrogated by administration of donor‐type dendritic cells (DCs) 2.

While modern antirejection treatments have concentrated on modifying the immune system of the recipient, relatively less work has been done on modifying that of the donor, with very few strategies that have been developed for the clinic. A pilot study perfusing human donor kidneys with anti‐CD45 antibodies to deplete donor passenger leukocytes before transplantation resulted in a reduced incidence of rejection 3.

Irradiation of rat livers or hearts to remove donor passenger leukocytes before transplantation can prolong graft survival 4, 5, 6. Murine islet allografts survived in the long term when treated more specifically with an anti‐DC antibody *in vitro* before transplantation 7. As donor‐derived professional antigen presenting cells are invariably MHC class II positive, attempts have been made to deplete this donor population to prolong graft survival. Early studies using a pan‐MHC class II antibody showed slight or no prolongation of graft survival 8, 9, 10, 11, 12, 13. However, these antibodies were not donor specific and therefore had to be perfused through the donor organ before transplantation rather than the simpler method of systemic administration to the recipient.

Studies using antidonor antibodies, including anti–MHC class II antibodies, have yielded mixed results. In a rat model, antidonor MHC class II antibody prolonged the survival of heart allografts 14 and induced donor‐specific tolerance of kidney grafts 15. The antibodies did not appear to be depleting, with the interaction between the antibodies and the donor passenger leukocytes seemingly imperative for this effect. In contrast, passive transfer of donor‐specific immune serum accelerated the rejection of donor‐type grafts 16.

Strategies such as parking or irradiation of donor organs are difficult or impossible to translate to the clinic. We aim to use an antidonor MHC class II antibody to deliver a toxin to donor MHC class II–expressing cells, to delete this population in a clinically applicable manner. The use of an immunotoxin, more commonly used in cancer therapy, allows targeted cell death without the need for complement activation or antibody‐dependent cell‐mediated cytotoxicity associated with the use of antibodies, and the inflammation that this may cause, which may promote alloreactivity. We have used the plant toxin gelonin, a ribosome inactivating protein found in the plant *Gelonium multiforum*, which causes cell death by inhibiting protein synthesis.

An MHC class II immunotoxin has previously been used in a pancreas allograft model 17, but like the early experiments with anti–MHC class II antibodies, this antibody was not donor specific and therefore had to be perfused through the pancreas before transplantation. The limited exposure to the immunotoxin, and at a low temperature, may have been responsible for its lack of efficacy.

In this study, we used an antibody against a single donor MHC class II molecule conjugated to gelonin (anti–I‐A^k^ gelonin immunotoxin, abbreviated as I‐A^k^‐gelonin) to deplete the MHC class II^+^ donor passenger leukocyte population in a fully mismatched mouse model of kidney transplantation, to investigate the effect of this treatment on graft survival.

## Materials and Methods

### Animals

All animals were between the age of 8 and 12 weeks and used in accordance with the Animals (Scientific Procedures) Act of 1986. Female C57BL/6 × CBA F1 (H‐2^b/k^), FVB (H‐2^q^), C57BL/6 (H‐2^b^), CBA.Ca (H‐2^k^), and BALB/c (H‐2^d^) mice were purchased from Harlan Limited (Bicester, UK). C57BL/6 mice expressing the enhanced yellow fluorescent protein (EYFP) transgene on all cells (rosa26 promoter knockin, abbreviated as BL/6 EYFP) and BL/6 EYFP × CBA F1 mice were bred in‐house. All mice were kept under specific pathogen‐free conditions.

### I‐A^k^‐gelonin immunotoxin preparation

A mouse anti‐mouse MHC class II I‐A^k,r,f,s^ IgG2a monoclonal antibody (Clone 10‐3.6.2, BioXCell, West Lebanon, NH) was digested into F(ab′)_2_ and F_c_ fragments using an F(ab′)_2_ preparation kit (Pierce [Thermo Fisher Scientific], Cramlington, UK) according to the manufacturer's instructions. The F(ab′)_2_ fragments were conjugated to gelonin (Enzo Life Sciences, Exeter, UK) as previously described 18. Briefly, the F(ab′)_2_ fragments were modified with 3‐(2‐pyridyldithio) propionic acid *N*‐hydroxysuccinimide ester (Sigma, Gillingham, UK) and incubated with a 5‐fold molar excess of gelonin, modified with 2‐iminothiolane (Sigma), for 20 hours before the reaction was terminated with 2 mmol/L iodoacetamide (Sigma). Unconjugated gelonin and F(ab′)_2_ fragments were removed via passage through a HiTrap rProtein A FF column (SLS, Nottingham, UK) and a HiTrap Blue HP column (SLS), respectively.

### In vitro killing assay

Single‐cell suspensions were obtained from spleens harvested from CBA and BALB/c mice. Red blood cells were lysed and the splenocytes plated at 2 × 10^6^ cells/mL in a 48‐well plate, with either unconjugated antibody, unconjugated F(ab′)_2_, or I‐A^k^‐gelonin, all at a concentration of 400 nmol/L. After 72 hours in culture at 37°C, 5% CO_2_, cells were removed for flow cytometry. Cells were stained with anti–I‐E^k^‐PE (clone 14‐4‐4S, eBioscience, Hatfield, UK), which recognizes the other MHC class II molecule on target cells so that binding of I‐A^k^‐gelonin to the target cells would not hinder the detection of MHC class II^+^ cells and a live/dead cell stain (Life Technologies, Paisley, UK), used according to the manufacturer's instructions. Cells were acquired using a FACScalibur flow cytometer (BD, Oxford, UK) and analyzed using Cellquest software version V3.3 (BD).

### In vivo killing assay

The effectiveness of the I‐A^k^‐gelonin in killing I‐E^K+^ cells *in vivo* was tested. Single‐cell suspensions were obtained from spleens harvested from BL/6 EYFP × CBA F1 mice. Red blood cells were lysed, and the splenocytes were injected intravenously into recipient mice (one spleen per recipient). I‐A^k^‐gelonin was then injected intravenously into these mice. Spleens of recipient mice were harvested 20 hours later, and flow cytometry was performed as described here earlier, using only anti–I‐E^k^‐PE antibody.

### Organ transplantation

Mouse renal transplantation was performed as described previously 19. I‐A^k^‐gelonin was given intravenously on the day of transplant. The recipient left native kidney was removed at the time of transplantation. The remaining right native kidney was removed 1 week after transplantation, leaving the donor graft life sustaining. Before and at regular intervals after the second native nephrectomy, blood samples were obtained and measurements of blood urea nitrogen (BUN) were made to monitor graft function, using Infinity Urea (Microgenics [Thermo Fisher Scientific], Hemel Hempstead, UK) according to the manufacturer's instructions. Heart transplantation was performed as described previously 20. Rejection of the transplanted heart was determined by palpation and confirmed by visual inspection. Skin transplantation was performed as described previously 21.

### Histology

Periodic acid–Schiff staining was performed on 2‐μm‐thick paraffin sections as previously described 22.

### Immunohistochemistry

Immunohistochemistry was performed on 5‐μm‐thick frozen sections as previously described 23 using an anti‐Foxp3 antibody (clone FJK‐16s, eBioscience).

### Donor‐specific alloantibody detection

Serum from recipient mice were used for the detection of donor‐specific alloantibodies as previously described 24.

### Enzyme‐linked immunospot assay

Enzyme‐linked immunospot (ELISpot) assays were performed as previously described 24, 25 using splenocytes harvested from heart transplant recipients 10 days posttransplantation. Results are expressed as number of spots per 2 × 10^5^ responder cells.

### Statistical analysis

Unpaired two‐tailed Student's t‐tests were used for all results except survival, which was tested by the log rank‐sum test. Values are expressed as mean ± standard error of mean.

## Results

### I‐A^k^‐gelonin immunotoxin kills target cells in a specific manner in vitro

**Figure 1:**
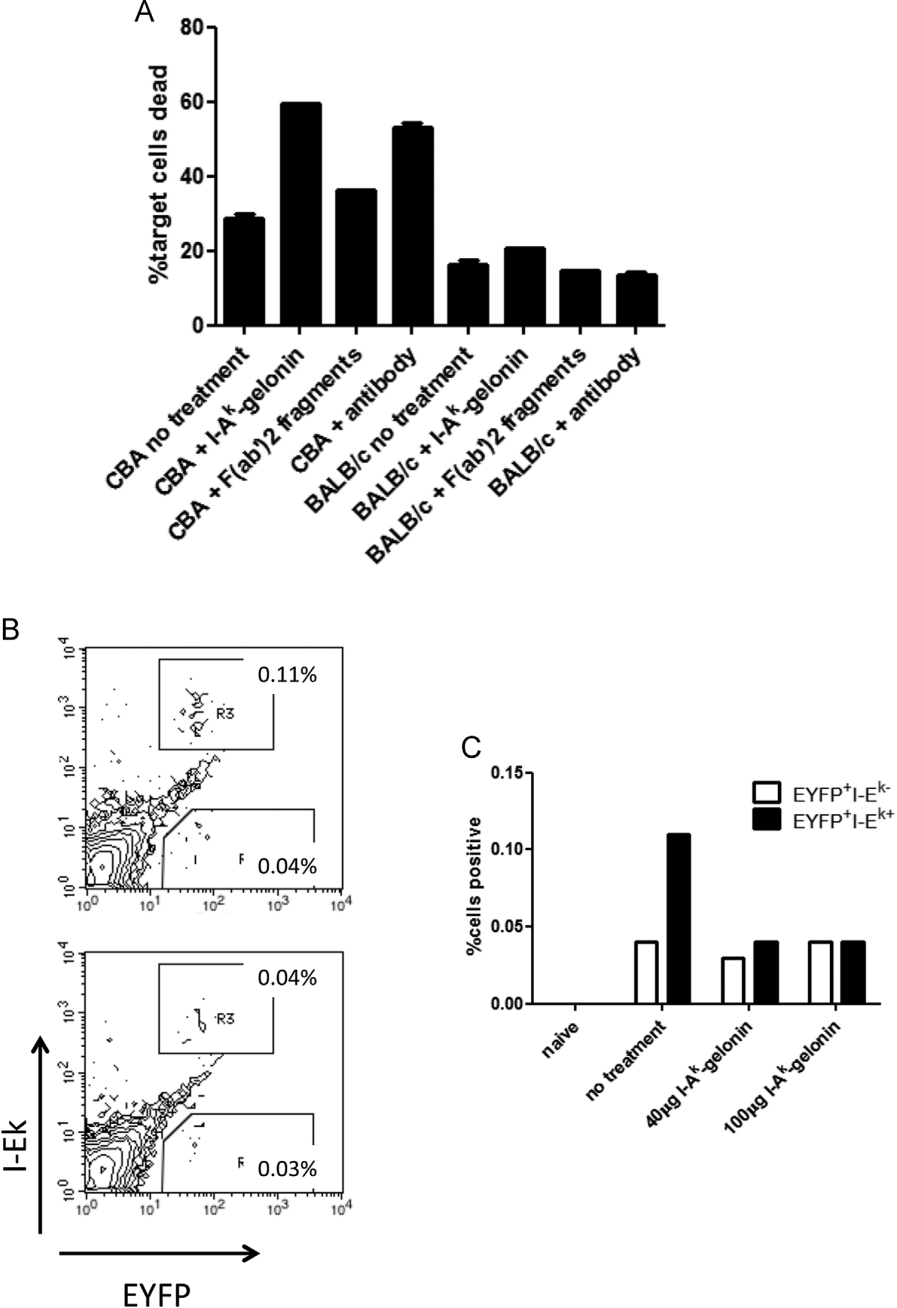
(A) CBA splenocytes, which express the target MHC class II molecule, and BALB/c splenocytes, which do not, were incubated with either I‐A^k^‐gelonin, unconjugated F(ab′)_2_ fragments, or unconjugated antibody. I‐A^k^‐gelonin and unconjugated antibody doubled the percentage of dead CBA cells after 72 hours of incubation, while unconjugated F(ab′)_2_ fragments had no effect. BALB/c cells were not killed. (B, C) Immunotoxin killing of target cells *in vivo*. BL/6 EYFP × CBA F1 splenocytes were injected into recipient mice with either no treatment (top FACS plot, B), 40 μg I‐A^k^‐gelonin (bottom FACS plot, B), or 100 μg I‐A^k^‐gelonin. The target I‐E^k^–expressing EYFP^+^ splenocyte population decreased after administration of I‐A^k^‐gelonin, while the nontarget I‐E^k‐^EYFP^+^ did not (C).

Incubation of CBA splenocytes (I‐A^k^ expressing) with I‐A^k^‐gelonin for 72 hours resulted in a doubling of cell death in the MHC class II^+^ population, demonstrating its effectiveness *in vitro* (Figure 1A). The killing was specific as it had no effect non–I‐A^k^–expressing BALB/c cells.

Unconjugated F(ab′)_2_ fragments had little effect on the death rate, while whole antibody increased the percentage of dead cells to a similar level seen with I‐A^k^‐gelonin.

### Target cells are killed by I‐A^k^‐gelonin immunotoxin in vivo

BL/6 EYFP × CBA F1 mice were used as splenocyte donors as they express both the EYFP protein, which allows tracking of donor cells in recipient mice, and the MHC class II molecule I‐A^k^ (inherited from the CBA parent) that is recognized by the I‐A^k^‐gelonin immunotoxin. Splenocyte injection was used instead of kidney transplantation here because it results in a higher number of cells that can be tracked, giving a more accurate dose titration. Splenocytes were injected into recipients with or without I‐A^k^‐gelonin treatment given intravenously, and 1 day later, their spleens were harvested for flow cytometry. This experiment was repeated several times with different doses of I‐A^k^‐gelonin. One representative experiment is shown in Figures 1B and 1C. After injection of splenocytes alone, 0.11% of cells express both EYFP and I‐E^k^. After injection of 40 or 100 μg of I‐A^k^‐gelonin, this population was reduced to 0.04%. The donor‐derived nontarget population (EYFP^+^ but negative for I‐E^k^) was not affected by I‐A^k^‐gelonin injection, at 0.03% and 0.04% with or without immunotoxin injection, respectively. The dose of 40 μg I‐A^k^‐gelonin was chosen for use in the kidney transplant study.

### I‐A^k^‐gelonin immunotoxin prolongs survival of kidney and heart allografts

**Figure 2:**
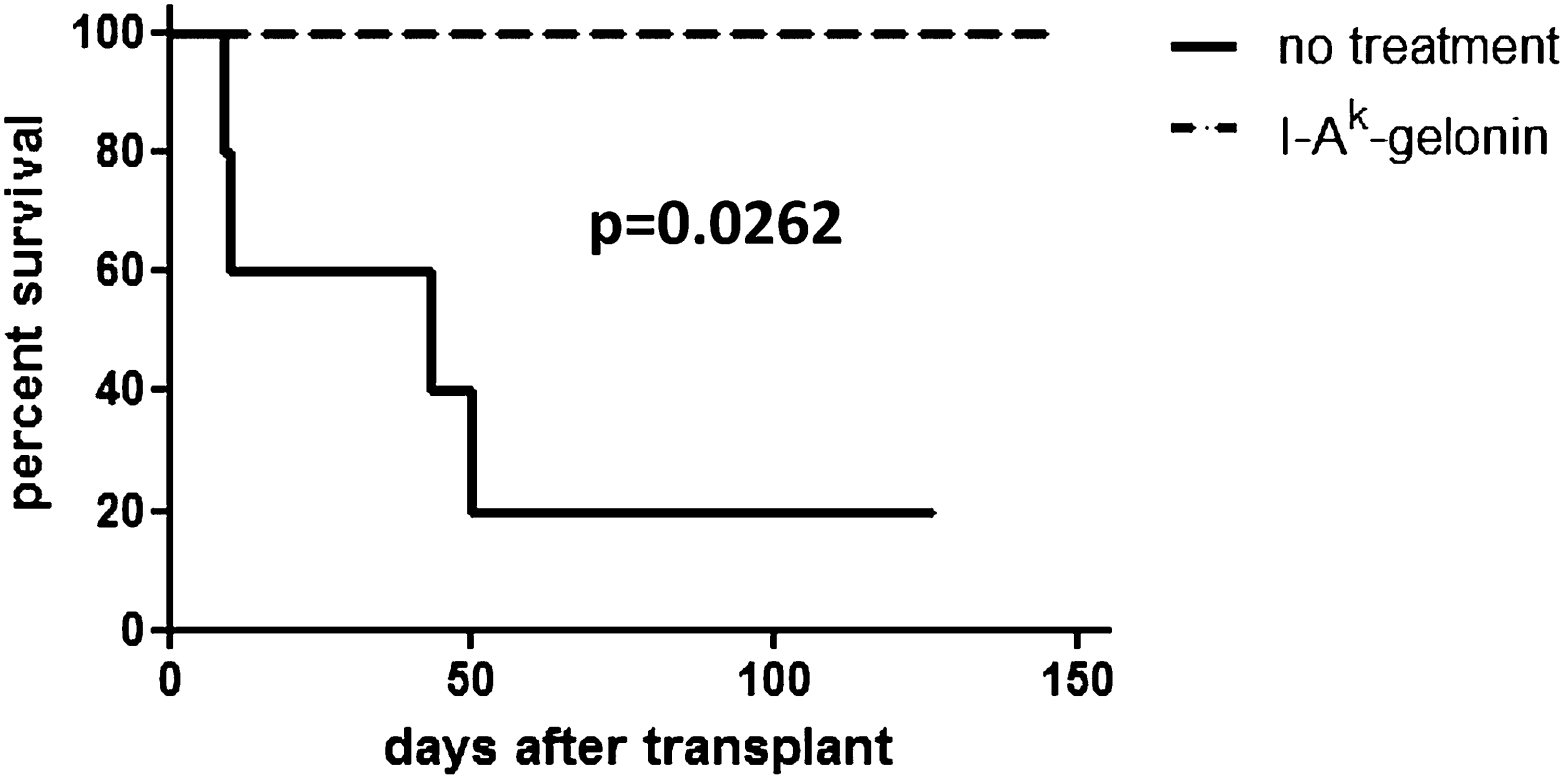
**Survival of FVB recipients of C57BL/6 × CBA F1 kidneys, with or without treatment with 40 μg I‐A^k^‐gelonin**. Without treatment, mice had a median survival time of 43 days (n = 5). With treatment, all mice survived long ‐term (n = 4) (median survival time >100 days, p = 0.0262).

**Figure 3:**
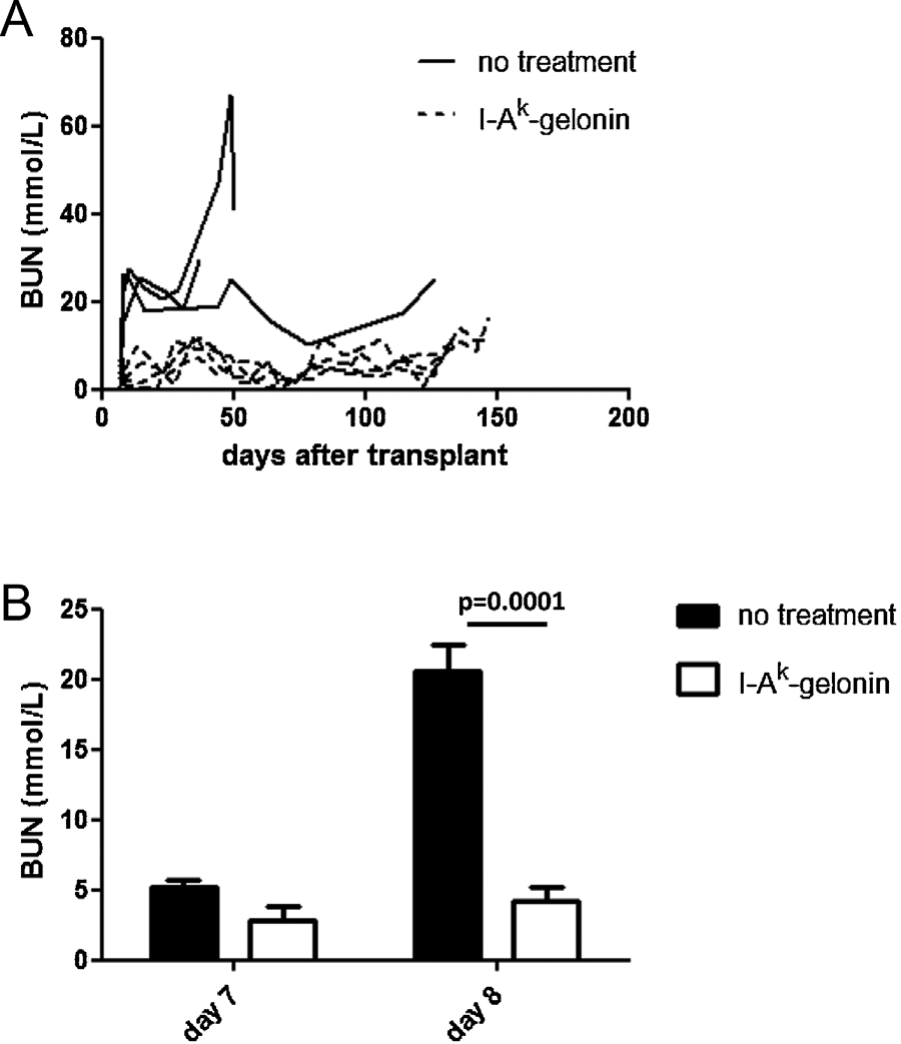
(A) BUN of FVB recipients of C57BL/6 × CBA F1 kidneys, with or without treatment with 40 μg I‐A^k^‐gelonin. (B) Comparison of BUN at day 7 posttransplantation, before the second native nephrectomy, and at day 8 posttransplantation, 1 day after the second native nephrectomy, when the graft becomes life‐sustaining. A considerable increase in the BUN of the no‐treatment group was seen but not in the I‐A^k^‐gelonin–treated group (p = 0.0001 at day 8).

To evaluate the effect of depleting donor MHC class II^+^ cells on graft survival, target antigen I‐A^k^–expressing C57BL/6 × CBA F1 kidneys were transplanted into fully MHC mismatched FVB recipients that do not express the target antigen I‐A^k^. In naive recipients, 40% of grafts were rejected acutely, with 40% undergoing chronic rejection and the remaining 20% surviving long term (median survival time 43 days; Figure 2). Graft rejection was accompanied by a decline in graft function resulting in an increase in BUN (Figure 3A). After one dose of 40 μg I‐A^k^‐gelonin, administered intravenously on the day of transplantation, 100% of grafts survived long term (Figure 2). BUN values in this group remained very low for the duration of the experiment, at all times remaining lower than the long‐term survivor in the control group (Figure 3A). The difference in graft function as measured by BUN is shown more clearly by comparing BUN at day 7, before the second native kidney of the recipient is removed, and at day 8, 1 day after the second nephrectomy when the graft becomes life sustaining. A sharp increase was seen in the control group, with BUN rising from 5.16 ± 0.54 mmol/L at day 7 to 20.64 ± 1.77 mmol/L at day 8, while the values for the treated group were 2.8 ± 1.03 and 4.2 ± 1.03, respectively (no significant difference at day 7, p = 0.0001 at day 8; Figure 3B).

**Figure 4:**
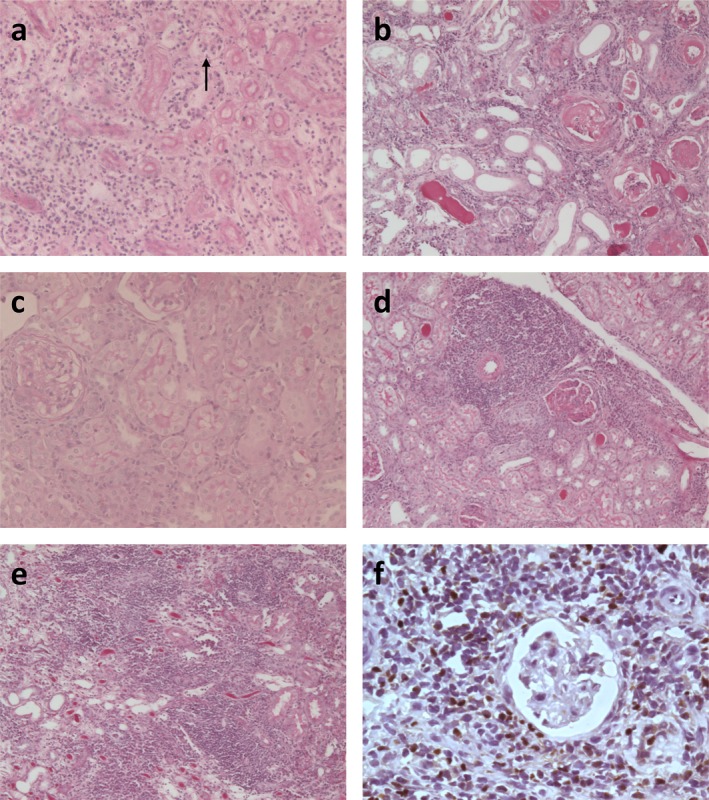
**Periodic acid–Schiff staining of sections of kidney allografts**. (A) Untreated kidney allograft rejected at day 9 posttransplantation, showing mononuclear cell infiltration, including tubulitis (arrow), and tubular necrosis (×200 magnification). (B) Representative examples of histological changes seen in donor grafts in untreated recipients. Three donor grafts were examined histologically from recipients that did not reject their grafts acutely. Interstitial fibrosis and tubular atrophy were seen (×100 magnification). (C) I‐A^k^‐gelonin–treated kidney allograft at day 147 posttransplantation, showing back‐to‐back tubules and little damage to the kidney (×200 magnification). (D) Tertiary lymphoid organ formation in I‐A^k^‐gelonin–treated kidney allograft (×100 magnification). (E) Tertiary lymphoid organs are much less well defined in long‐term surviving untreated kidney allograft (×100 magnification). (F) Foxp3^+^ cells in tertiary lymphoid organs of I‐A^k^‐gelonin–treated kidney allografts (×400 magnification).

Donor grafts from control mice that rejected their grafts at early time points (days 9 and 10 posttransplantation) showed mononuclear cell infiltration, including tubulitis, and tubular necrosis (Figure 4A). Of the control mice that did not reject acutely, donor grafts showed signs of chronic tubular and glomerular damage consistent with interstitial fibrosis and tubular atrophy (Figure 4B). Donor grafts from I‐A^k^‐gelonin–treated recipients, in contrast, showed well‐preserved tubules and glomeruli with little sign of damage (Figure 4C). Mononuclear cell infiltration was in the main restricted to tertiary lymphoid organs (TLOs) within the donor graft (Figure 4D). In the long‐term survivor of the control group, the TLOs were less defined and there was more infiltrate outside of these structures (Figure 4E). Foxp3 staining of donor grafts from I‐A^k^‐gelonin–treated recipients revealed the presence of regulatory T cells concentrated within these TLOs (Figure 4F), suggesting regulation of the alloimmune response.

I‐A^k^‐gelonin was also tested in a heart transplantation model, using the same donor–recipient strain combination. Treatment of recipients with I‐A^k^‐gelonin led to a slight prolongation of heart graft survival from a median survival time of 17.5 to 31.5 days (n = 4).

To ensure that the effect seen is donor specific, I‐A^k^‐gelonin was administered to FVB recipients of C57BL/6 hearts, which do not express I‐A^k^ and whose passenger leukocytes should therefore not be depleted by the immunotoxin. Treatment of recipients with I‐A^k^‐gelonin had no effect on graft survival (median survival time 11 days vs 10 days for treated and control groups, respectively, n = 3).

### Prolonged survival of challenge skin grafts expressing donor MHC molecules regardless of immunotoxin specificity

**Figure 5:**
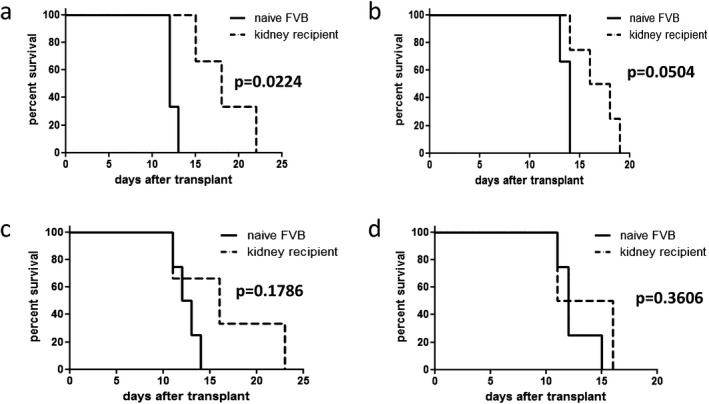
**Survival of challenge skin grafts**. Skin from C57BL/6 × CBA F1 donors (A), C57BL/6 (B), and CBA (C) parental strain donors and third party BALB/c donors (D) was transplanted onto FVB recipients of C57BL/6 × CBA F1 kidney transplants and I‐A^k^‐gelonin, around 100 days posttransplantation, and naive FVB mice. C57BL/6 × CBA F1 donor skin showed prolonged survival [median survival time of 18 vs 12 days in kidney recipients vs naive, respectively, p = 0.0224 (A)]. C57BL/6 and CBA skin allografts also had slightly prolonged survival in kidney recipients compared with naive mice, with median survival times of 17 versus 14 days (B) and 16 versus 12.5 days (C), respectively, but this did not reach statistical significance. No extended survival of third‐party BALB/c skin grafts was observed with median survival times of 13.5 days in kidney recipients compared with 12 days in naive mice (D).

FVB recipients of C57BL/6 × CBA F1 kidney transplants treated with I‐A^k^‐gelonin were given challenge skin grafts at around 100 days posttransplantation. The original donor strain, C57BL/6 × CBA F1, the two parental strains C57BL/6 and CBA (not expressing and expressing I‐A^k^ recognized by the immunotoxin, respectively), as well as third‐party BALB/c mice were used as donors. C57BL/6 × CBA F1 donor skin showed prolonged survival (median survival time of 18 vs 12 days in kidney recipients vs naive, respectively, p = 0.0224; Figure 5A). C57BL/6 and CBA skin allografts also had slightly prolonged survival in kidney recipients compared with naive mice, with median survival times of 17 versus 14 days (Figure 5B) and 16 versus 12.5 days (Figure 5C), respectively, but this did not reach statistical significance. No extended survival of third‐party BALB/c skin grafts was observed (Figure 5D).

### Decreased donor‐specific alloantibody levels in I‐A^k^‐gelonin–treated kidney recipients

**Figure 6:**
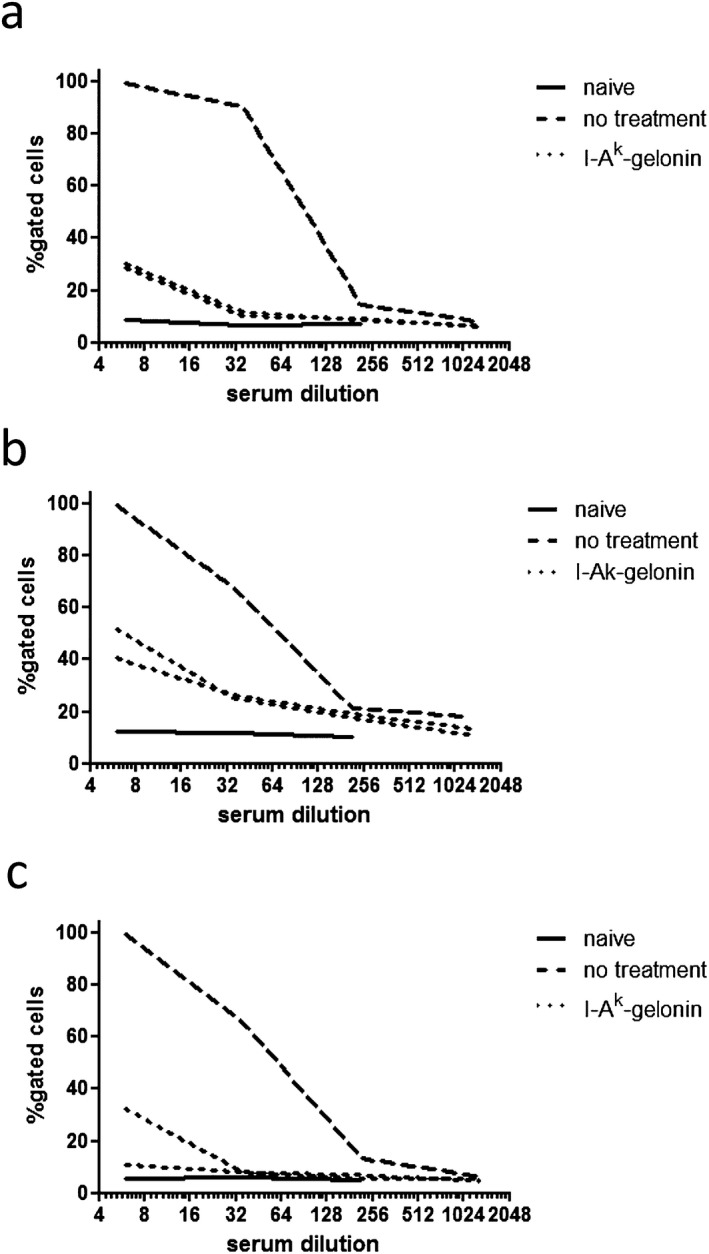
**Donor‐specific alloantibody detection in serum from kidney allograft recipients**. Splenocytes from C57BL/6 × CBA F1 (A), C57BL/6 (B), and CBA (C) mice were incubated with serum from untreated and I‐A^k^‐gelonin–treated kidney recipients taken at 100 days posttransplantation. Naive FVB serum was used as a control. An anti‐IgG FITC then detected the percentage of target splenocytes that had bound donor‐specific alloantibodies from the serum. The percentages of these splenocytes positive for alloantibody from serum from I‐A^k^‐gelonin–treated kidney allograft recipients were much lower than for serum from the untreated recipient. The percentages of splenocytes that bound alloantibody decreased with dilution of serum.

Donor‐specific antibody titers were measured in serum taken from recipients 100 days after transplantation. Much higher titers of donor‐specific antibody were seen in serum from the untreated recipient compared with the two recipients treated with I‐A^k^‐gelonin when either the original donor strain C57BL/6 × CBA F1 or either parental strain was used as target cells (Figure 6).

### Alloimmune response after I‐A^k^‐gelonin treatment

Heart grafts and spleens from I‐A^k^‐gelonin–treated or control FVB recipients of C57BL/6 × CBA F1 heart transplants were harvested 10 days after transplantation to quantify the alloimmune response to determine if it was affected by immunotoxin treatment.

Sections of the transplanted heart were stained for Foxp3 expression. The number of Foxp3^+^ cells was slightly but not significantly higher in I‐A^k^‐gelonin–treated grafts compared with controls (5.35 ± 2.16 vs 4.89 ± 1.25 cells per high‐power field, respectively).

**Figure 7:**
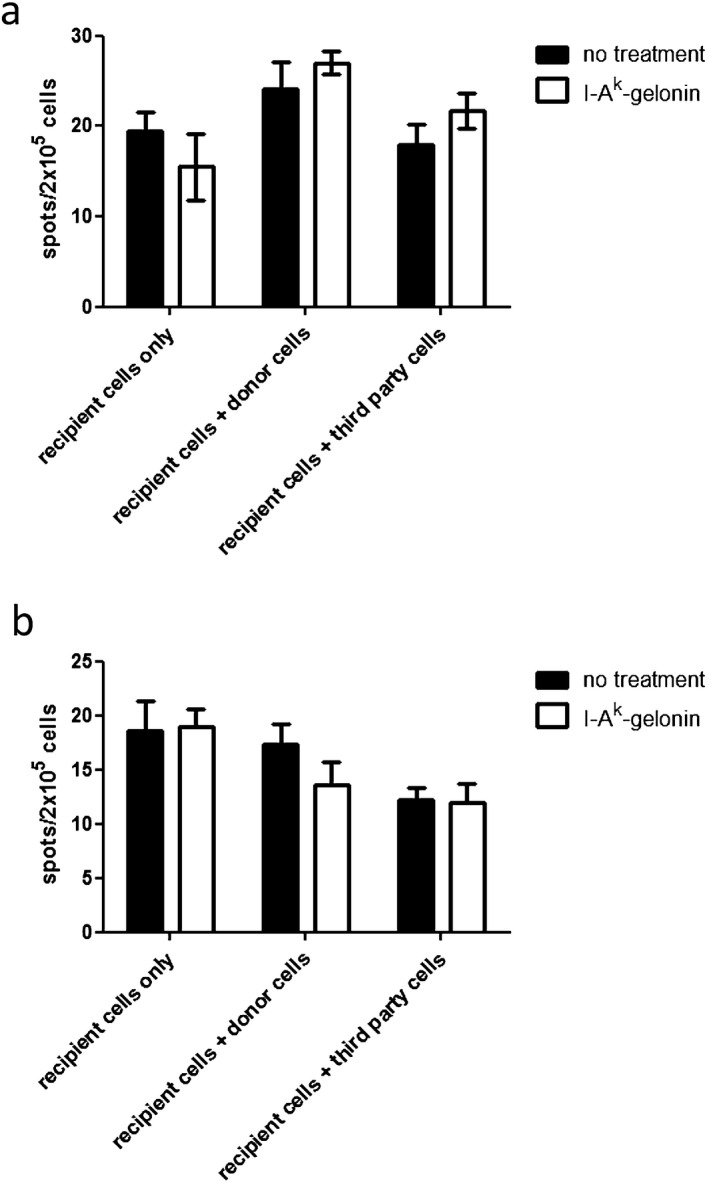
**IFNγ production by splenocytes from I‐A^k^‐gelonin–treated or control heart allograft recipients harvested 10 days after transplantation**. Recipient splenocytes were stimulated with either whole donor splenocytes (A), to measure the total alloresponse, or lysed donor splenocytes (B), to measure the indirect pathway only. No differences between control and treated groups were found.

Splenocytes from treated and control FVB recipients of C57BL/6 × CBA F1 heart transplants were used in ELISpot assays to detect interferon‐γ (IFNγ) production in response to either alloantigen presented via the indirect pathway only (using lysed donor splenocytes as stimulator cells) or alloantigen presented by both the direct and indirect pathways (using intact donor splenocytes as stimulator cells). Because DCs were not removed from the recipient splenocyte preparation, an IFNγ response was seen even without the addition of donor cells as stimulators but with no differences between control and I‐A^k^‐gelonin–reated splenocytes (19.5 ± 2.08 and 15.5 ± 3.73 spots/2 × 10^5^ cells, respectively). This was probably due to the indirect pathway, because by the time point chosen (day 10 posttransplantation) we would expect most donor passenger leukocytes to have been killed. The addition of donor cells as stimulators resulted in an increase in IFNγ production through the direct pathway in both control and I‐A^k^‐gelonin–treated groups (24.17 ± 2.86 and 27 ± 1.28 spots/2 × 10^5^ cells, respectively), while no or very little increase was seen when third‐party cells were used as stimulators (17.92 ± 2.26 and 21.67 ± 1.94 spots/2 × 10^5^ cells, respectively) (Figure 7A). When using lysed stimulator cells, no increase in IFNγ production was seen, probably due to the fact that the indirect pathway was already in action within the recipient splenocyte population. In fact, a small reduction was seen when lysed stimulator cells were used (Figure 7B).

## Discussion

Given the importance of donor passenger leukocytes in the alloimmune response, it makes sense to develop strategies to deplete them to improve graft survival, such as using monoclonal antibodies or immunotoxins. Antibodies have the advantage of allowing specific depletion of the target cell populations and are already being routinely used in the clinic to deplete or modify recipient cells (e.g. alemtuzumab to deplete CD52^+^ cells), but antibodies cause cell death through complement activation or antibody‐dependent cell‐mediated cytotoxicity, both of which cause inflammation. When used to target donor cells, this has the potential to exacerbate the alloimmune response. Immunotoxins combine the specificity of antibodies with killing by toxin delivery, which may avoid these potential problems.

Although immunotoxins were developed as a cancer therapy and are much more commonly used in this setting, immunotoxins have been used in transplantation. An anti‐CD3 antibody combined with diphtheria toxin has been successfully used in primates to deplete recipient T cells 26. Several immunotoxins have also been used to deplete donor T cells to prevent graft‐versus‐host disease after bone marrow transplantation 27, 28.

Gelonin, the ribosome‐inactivating protein used here, is unable to enter cells in its unconjugated form. Instead, it relies on antibody internalization for delivery into the cytoplasm and therefore is highly specific. To avoid complement activation and antibody‐dependent cell‐mediated cytotoxicity, the antibody used here was digested to remove the F_c_ portion responsible for complement activation and binding to leukocytes, leaving only the antigen‐binding F(ab′)_2_ component.

The C57BL/6 × CBA F1 to FVB kidney transplant model was chosen to study the effect of I‐A^k^‐gelonin on graft survival due to the range of outcomes seen, allowing the immunotoxin's effect on both acute and chronic rejection and on long‐term survival to be studied. The use of F1 donors also enabled us to use either parental strain as challenge graft donors.

Long‐term graft survival was seen in treated kidney recipients, while heart recipients showed a modest prolongation of survival. This may be because heart grafts are more immunogenic than kidney grafts. As the immunotoxin would not have depleted donor passenger leukocytes immediately, the initial exposure of the immune system of the recipient may have resulted in the rejection of the heart grafts, albeit at a delayed tempo. Kidney grafts however, are more “tolerogenic,” and the reduced immune response resulting from the immunotoxin treatment may have been sufficient to induce indefinite graft survival. Long‐term acceptors of kidney grafts demonstrated prolonged survival of donor‐type skin grafts, but their survival was not indefinite. This is not surprising because skin is far more immunogenic than kidney grafts, and it is not unusual to observe split tolerance. In addition, skin‐specific antigens may also play a role here.

Tolerance was clearly not induced in the heart transplant model. If translated to the clinic, immunotoxin treatment would not be a means to induce indefinite graft survival but may be a useful adjuvant to prevent rejection. Because it is donor specific, the risk of opportunistic infections and malignancies should not be increased.

In the kidney transplant model, we did observe indefinite graft survival. It has been shown that donor passenger leukocytes are essential for tolerance induction 29, 30. Our observation is not in discordance with this. First, the depletion was not immediate. The reduced number (but not absence) of donor passenger leukocytes may have resulted in an immune reaction that was not fully effective, leading to a dampened immune response. Second, in the kidney graft model, the graft itself may have “tolergenic” properties to induce long‐term survival independent of donor passenger leukocytes.

The indefinite survival induced in kidney transplant recipients suggests that the immunotoxin itself is unlikely to have any detrimental effects on the graft and can protect it from the rejection process. Mouse endothelial cells can upregulate MHC class II following transplantation 31. Therefore, donor grafts could be vulnerable to immunotoxin‐mediated depletion. However, we have not observed any detrimental effects. This may be due to the short half‐life of the immunotoxin, which may have been removed from the circulation by the time MHC class II is expressed on endothelial cells. The constitutive expression of MHC class II molecules by human endothelial cells may hamper the translation of this technique to the clinic. Strategies to selectively direct the immunotoxin toward donor passenger leukocytes while sparing endothelial cells will be required. These are currently being developed in our laboratory.

Because the I‐A^k^‐gelonin depletes donor passenger leukocytes, it dampens the direct pathway of allorecognition. Due to this interference with the direct pathway of allorecognition, it might have been expected that the I‐A^k^‐gelonin would have an effect on acute rejection, which is thought to primarily be caused by the direct pathway, but no effect on chronic rejection, which is thought to be mediated mainly by the indirect pathway. However, I‐A^k^‐gelonin administration prevented both acute and chronic rejection and instead induced long‐term survival of the kidney grafts and extended the survival of challenge skin grafts. This may be due to the fact that the direct pathway of allorecognition is the first to appear and is the bigger response in terms of the percentage of T cells that can recognize foreign MHC class II directly [estimated at between 1% and 7% of T cells 32, 33]. The dampening of the adaptive immune response against the graft may allow this process to be controlled by cells such as regulatory T cells, which are mainly, if not all, of indirect specificity 34 and therefore should be unaffected by the I‐A^k^‐gelonin. The presence of Foxp3^+^ cells [which have previously been shown to be important in spontaneous acceptance of kidney allografts 35, 36] within the donor grafts after treatment with I‐A^k^‐gelonin is in agreement with this. However, there was very little difference between the number of Foxp3^+^ cells seen in heart grafts 10 days after transplantation in treatment and control groups. This may be because heart grafts are more immunogenic, and therefore any beneficial effect of the immunotoxin is not as obvious.

In an attempt to determine the effect of I‐A^k^‐gelonin on the direct and indirect pathways separately, ELISpot assays were performed in which either whole donor splenocytes were used, meaning antigen could be presented by both direct and indirect pathways, or lysed donor splenocytes were used, meaning antigen could be presented only by the indirect pathway 25. No differences were seen between I‐A^k^‐gelonin–treated and control groups. This may be due to the time point chosen (10 days posttransplantation), which may be too late to detect any effect of the I‐A^k^‐gelonin treatment. Also, the ELISpots were carried out on heart transplant recipients, which were less sensitive to I‐A^k^‐gelonin treatment.

Treatment with I‐A^k^‐gelonin did reduce the level of circulating donor‐specific antibodies in kidney recipients, presumably because of a reduction in the number of alloantigens available. However, the presence of circulating donor‐specific antibodies, albeit at lower levels, in I‐A^k^‐gelonin–treated recipients of kidney allografts suggests an active immune interaction with the graft, as does the presence of TLOs within these grafts. In a long‐term surviving DBA/2 to C57BL/6 model of kidney transplantation, the presence of donor‐specific antibodies does not affect graft function (unpublished information) and TLO formation is indirectly proportional to BUN 37. The evidence for an ongoing immune response without concomitant poor graft function or histological signs of rejection suggests a dynamic interaction between the regulatory and effector arms of the alloimmune response in I‐A^k^‐gelonin–treated recipients.

Our data suggest that depletion of donor passenger leukocytes, resulting in indefinite kidney graft survival and good graft function, can be achieved with a single treatment of an antidonor MHC class II immunotoxin administered to the recipient after transplantation. Current antirejection treatment is almost exclusively directed against the immune response of the recipient. The approach described here, directed specifically against cells of donor origin, opens a new, simple, and safe therapeutic avenue that can be used in conjunction with conventional therapy.

## Disclosure

The authors of this manuscript have no conflicts of interest to disclose as described by the *American Journal of Transplantation*.
